# UBL3 Interacts with PolyQ-Expanded Huntingtin Fragments and Modifies Their Intracellular Sorting

**DOI:** 10.3390/neurolint16060089

**Published:** 2024-10-22

**Authors:** Soho Oyama, Hengsen Zhang, Rafia Ferdous, Yuna Tomochika, Bin Chen, Shuyun Jiang, Md. Shoriful Islam, Md. Mahmudul Hasan, Qing Zhai, A. S. M. Waliullah, Yashuang Ping, Jing Yan, Mst. Afsana Mimi, Chi Zhang, Shuhei Aramaki, Yusuke Takanashi, Tomoaki Kahyo, Yoshio Hashizume, Daita Kaneda, Mitsutoshi Setou

**Affiliations:** 1 Department of Cellular and Molecular Anatomy, Hamamatsu University School of Medicine, 1-20-1 Handayama, Chuo-ku, Hamamatsu 431-3192, Shizuoka, Japan; oyamasoho@gmail.com (S.O.); hengsen19110@gmail.com (H.Z.); rafiaru22@gmail.com (R.F.); hhelibeyuna@gmail.com (Y.T.); chenbin19101@gmail.com (B.C.); shuyungoole@gmail.com (S.J.); shoriful.pharmacy21@gmail.com (M.S.I.); hasan.mahmudbio@gmail.com (M.M.H.); zhaiqing199404@gmail.com (Q.Z.); wali.rubmb10@gmail.com (A.S.M.W.); pingyashuang1989@gmail.com (Y.P.); yanjing20105@gmail.com (J.Y.); afsana.mimichem@gmail.com (M.A.M.); zhangchi07.pegasus@gmail.com (C.Z.); aramaki.shh@gmail.com (S.A.); kahyo@hama-med.ac.jp (T.K.); 2 Department of Neurosurgery, The Affiliated Hospital of Jiangnan University, Wuxi 214000, China; 3 Department of Orthopedic Surgery, Hamamatsu University School of Medicine, 1-20-1 Handayama, Chuo-ku, Hamamatsu 431-3192, Shizuoka, Japan; 4 First Department of Surgery, Hamamatsu University School of Medicine, 1-20-1 Handayama, Chuo-ku, Hamamatsu 431-3192, Shizuoka, Japan; nashimed1@gmail.com; 5 Quantum Imaging Laboratory, Division of Research and Development in Photonics Technology, Institute of Photonics Medicine, Hamamatsu University School of Medicine, 1-20-1 Handayama, Chuo-ku, Hamamatsu 431-3192, Shizuoka, Japan; kaneda@chojuken.net; 6 Choju Medical Institute, Fukushimura Hospital, Yamanaka-19-14 Noyoricho, Toyohashi 441-8124, Aichi, Japan; kyhashi@chojuken.net; 7 International Mass Imaging and Spatial Omics Center, Institute of Photonics Medicine, Hamamatsu University School of Medicine, 1-20-1 Handayama, Chuo-ku, Hamamatsu 431-3192, Shizuoka, Japan

**Keywords:** ubiquitin-like 3, huntington’s disease, polyglutamine, huntingtin protein, interaction, sorting

## Abstract

Background/Objectives: UBL3 (Ubiquitin-like 3) is a protein that plays a crucial role in post-translational modifications, particularly in regulating protein transport within small extracellular vesicles. While previous research has predominantly focused on its interactions with α-synuclein, this study investigates UBL3’s role in Huntington’s disease (HD). HD is characterized by movement disorders and cognitive impairments, with its pathogenesis linked to toxic, polyglutamine (polyQ)-expanded mutant huntingtin fragments (mHTT). However, the mechanisms underlying the interaction between UBL3 and mHTT remain poorly understood. Methods: To elucidate this relationship, we performed hematoxylin and eosin (HE) staining and immunohistochemistry (IHC) on postmortem brain tissue from HD patients. Gaussia princeps-based split-luciferase complementation assay and co-immunoprecipitation were employed to confirm the interaction between UBL3 and mHTT. Additionally, we conducted a HiBiT lytic detection assay to assess the influence of UBL3 on the intracellular sorting of mHTT. Finally, immunocytochemical staining was utilized to validate the colocalization and distribution of these proteins. Results: Our findings revealed UBL3-positive inclusions in the cytoplasm and nuclei of neurons throughout the striatum of HD patients. We discovered that UBL3 colocalizes and interacts with mHTT and modulates its intracellular sorting. Conclusions: These results suggest that UBL3 may play a significant role in the interaction and sorting of mHTT, contributing to the understanding of its potential implications in the pathophysiology of Huntington’s disease.

## 1. Introduction

Ubiquitin-like 3 (UBL3), a highly conserved ubiquitin-like protein present in eukaryotes, is also recognized as a membrane-anchored ubiquitin-fold protein [1]. In previous studies, we found UBL3’s role as a critical factor in the post-translational modification of proteins and its capacity to facilitate the sorting of proteins into small extracellular vesicles [2,3]. We hypothesized that UBL3 may be involved in the pathogenesis of neurodegenerative diseases by interacting with insoluble toxic proteins and altering their intracellular transport. Recently, we reported the interaction between UBL3 and alpha-synuclein (α-syn), demonstrating that Microsomal Glutathione S-Transferase 3 (MGST3) upregulates this interaction. These findings provide evidence of a connection between UBL3 and α-synucleinopathy, suggesting potential therapeutic implications [4,5,6].

Huntington’s disease (HD) is an autosomal-dominant neurodegenerative disorder characterized by a triad of mobility problems, psychiatric symptoms, and cognitive impairments [7,8]. HD is caused by the amplification of Cytosine–Adenine–Guanine (CAG) trinucleotide repeats (≥36) in the huntingtin (HTT) gene, with the causative agent being a mutated form of the multifunctional protein HTT [9,10]. The mutant form of HTT (mHTT) contains an expanded polyglutamine (polyQ) sequence, corresponding to the CAG gene amplification, which renders the protein toxic and leads to neuronal dysfunction and death [11,12]. It has been shown that this toxic mHTT interacts with various proteins and alters their intracellular transport [13,14].

The modification of intracellular transport by mHTT occurs through several mechanisms, including aggregation, direct inhibition, and altered interactions. For example, the interaction between small ubiquitin-like modifier (SUMO) proteins and the N-terminal region of mHTT can promote its aggregation [15,16]. Additionally, the N-terminal of mHTT interacts with E6-associated protein ubiquitin ligase, which facilitates the proteolytic degradation through the ubiquitin–proteasome system, thereby inhibiting their aggregation and toxicity [17]. Moreover, mHTT can directly inhibit protein synthesis and slow ribosomal translocation, affecting the production and movement of proteins within cells [18].

However, it remains unclear how UBL3 interacts with mHTT and whether it influences the intracellular transport of mutant huntingtin. Therefore, we hypothesized that UBL3 may be involved in the pathological processes associated with the delivery and accumulation of mHTT in the neurons of Huntington’s disease (HD) patients. In this study, we primarily investigated the distribution of UBL3 in postmortem brain tissue, specifically the striatum of HD patients, and examined the interaction between UBL3 and polyQ-expanded huntingtin fragments. We then explored UBL3’s influence on the intracellular sorting of mHTT and assessed their colocalization within the cells.

## 2. Materials and Methods

### 2.1. Human Postmortem Brain Samples

The Huntington’s disease (HD) samples used in this study were obtained from the Choju Medical Institute of Fukushimura Hospital. After sampling, the tissues were frozen on a copper plate cooled with dry ice and stored at −80 °C. HD was clinically, pathologically, and genetically diagnosed according to the current diagnostic criteria [19]. Genetic testing of the number of CAG repeats in the HTT gene were outsourced to SRL, Inc., Nagoya, Japan. The controls were postmortem brain tissue specimens from patients diagnosed with non-Hodgkin’s lymphoma, obtained from the Department of Regenerative Infection Pathology at Hamamatsu University School of Medicine. The clinical information of each patient is shown below in Table 1. The polyQ repeat number represents the pathogenic and normal alleles of the HTT gene.

### 2.2. Antibodies

The antibodies used were the anti-polyglutamine-expansion disease marker antibody clone 5TF1-1C2 (MERCK, Darmstadt, Germany, MAB1574, 1:1000 dilution), anti-MYC antibody (MBL, Tokyo, Japan; M1932, 1:1000 dilution), anti-Flag antibody (MERCK, Darmstadt, Germany, F7425-.2MG, 1:1000 dilution), anti-Beta-Actin antibody (CST, Danvers, MA, USA 8H10D10, 1:1000 dilution), biotinylated anti-rabbit immunoglobulin G (Vector Laboratories, Newark, CA, USA, BA-1000, 1:1000 dilution), horseradish peroxidase (HRP)-conjugated anti-rabbit secondary antibody (CST, 7074, 1:5000 dilution), HRP-conjugated anti-mouse secondary antibody (eBioscience, San Diego, CA, USA, 18-8817, 1:5000 dilution), Alexa Fluor 488-conjugated anti-rabbit secondary antibody (Invitrogen, Waltham, MA, USA, A11008, 1:500 dilution), and Alexa Fluor 647-conjugated anti-mouse secondary antibody (Invitrogen, A21235, 1:500 dilution). The polyclonal anti-UBL3 antibody was made in our laboratory (Supplementary Figure S1), using UBL3 without the C-terminal CCVIL as the antigen.

### 2.3. Hematoxylin and Eosin (HE) Staining

The sections were immersed in hematoxylin solution for 5–10 min, rinsed in running tap water, and differentiated in a 1% acid alcohol solution for 1–2 s before being blued in 0.2% ammonia water or lithium carbonate solution for 1–2 min. After a final rinse in tap water, the sections were stained in eosin Y solution for 1–2 min, briefly rinsed in 95% ethanol, dehydrated through an ascending ethanol series (70%, 95%, 100%), and cleared in xylene. Finally, the sections were mounted on slides, and images were captured using a Nano-zoomer2.0-HT slide scanner (Hamamatsu Photonics, Hamamatsu, Japan).

### 2.4. Immunohistochemistry (IHC) Staining

For IHC staining examination, the sections were deparaffinized and rehydrated. Subsequently, they were treated with 3% H_2_O_2_ for 10 min to block endogenous peroxidase activity. Antigen retrieval was performed using 10 mM citrate buffer pH 9.0 for 1 min at 105 °C in an autoclave. Afterward, the sections were blocked with 1% Bovine Serum Albumin (BSA, MERCK) for 1 h at room temperature. The sections were then incubated with primary antibodies, followed by 1 h incubation with a biotinylated secondary antibody (Vector Laboratories) at room temperature. Subsequent to the secondary antibody incubation, the slides were incubated in an ABC solution (Vectastain Elite ABC Kit, Vector Laboratories) for 1 h. Finally, the sections were exposed to a 3′3-diaminobenzidine (DAB) solution for 5 min. After DAB exposure, the slides were counterstained with hematoxylin, following by rinsing and dehydration. Coverslips were mounted on the slides, and images were captured using a Nanozoomer2.0-HT slide scanner (Hamamatsu Photonics, Hamamatsu, Japan).

### 2.5. Plasmids Construction

For the NGluc-UBL3 and NGluc-UBL3∆5 plasmids, which respectively contain the N-terminal region of Gaussia princeps luciferase (Gluc) sequence-tagged UBL3 with or without the deletion of the “CCVIL” amino acid sequence in the C-terminal region of UBL3, along with Flag-UBL3 and Flag-UBL3∆5 plasmids, were previously utilized in our laboratory [4]. To construct the plasmid for the C-terminal region of Gluc sequence-tagged expanded polyQ-containing N-terminal HTT fragments (nHTTpolyQ78-CGluc), the coding sequence spanning from codons 1 to 156 of HTT, including HD exon1 and 78 CAG repeats, was inserted in the frame preceding the CGluc sequence in the pCI vector. This insertion was performed between the Xho I site and the Mlu I site. For the MYC-nHTTpolyQ78 plasmid, it was generated by PCR amplification using following primers: 5′-CCGCTCGAGATGGCGACCCTGGAAAAGC-3′ (forward) and 5′-GCCTCTAGATTA ACATATTGTCAGACAATGATTCACACGG-3′ (reverse). Subsequently, the fragment obtained was inserted into the pcDNA3-MYC vector after the MYC sequence, following digestion by XhoI and XbaI. The fragments of NGluc-UBL3, NGluc-UBL3∆5, and nHTTpolyQ78-CGluc were tagged with an immunoglobulin kappa secretory signal sequence after the start codon. This tag facilitated the secretion of the expressed fragments or interacting complexes into the cell culture medium.

### 2.6. Cell Culture and Transfection

Human embryonic kidney (HEK) 293 cells (RIKEN cell Bank, Tsukuba, Ibaraki, Japan) were cultured in Dulbecco’s modified Eagle’s medium (DMEM, Thermo Fisher Scientific, Waltham, MA, USA) supplemented with 10% fetal bovine serum (FBS) (Sigma-Aldrich, St. Louis, MI, USA). The cells were cultured at 37 °C in a 5% CO_2_ humidified incubator. Until the cells reached 60–80% confluence, they were transiently transfected with cDNA plasmids using Lipofectamine 2000 transfection reagent (Thermo Fisher Scientific, Waltham, MA, USA) diluted in Opti-MEM (Thermo Fisher Scientific, Waltham, MA, USA), following the manufacture’s recommendations.

### 2.7. Gaussia Princeps-Based Split-Luciferase Complementation Assay

Culture media (CM) from the cells were collected and centrifuged at 1200 rpm for 5 min to remove cell debris. The cells were lysed with 1% Triton X-100 (Sigma-Aldrich, St. Louis, MI, USA) and centrifuged at 13,000 rpm for 5 min to obtain the supernatant. After adding 17 μg/mL of Coelenterazine (C_26_H_21_N_3_O_3_, Cosmo Bio, Kyodo, Japan), which was diluted in Opti-MEM, the luciferase activity was immediately measured using the microplate reader (BioTek, Winooski, VT, USA). The luciferase activity of untreated DMEM (10% FBS) was used as a background.

### 2.8. Immunoprecipitation (IP)

After a 72-h incubation, the cells were washed and collected by scraping into ice-cold phosphate-buffered saline (PBS), pelleted by centrifugation at 5000 rpm for 3 min at 4 °C, and lysed with 1% Triton buffer (50 mM Tris-HCl [pH 7.4], 100 mM NaCl, and 1% [*v*/*v*] Triton X-100) for 30 min on ice. Cell debris and unbroken cells were removed by centrifugation at 15,000 rpm for 20 min at 4 °C. Using the BCA assay, the protein content of the supernatant was determined in accordance with the manufacturer’s instructions. A total of 50 μL of anti-Flag tag antibody magnetic beads (FUJIFILM Wako Pure Chemical Corporation, Osaka, Japan) were incubated with supernatants containing 500 μg of total protein for 10 h at 4 °C. After washing the beads three times with ice-cold wash buffer (50 mM Tris-HCl [pH 7.4], 100 mM NaCl), the beads were then mixed with 50 μL of 2-mercaptoethanol (−2× sodium dodecyl sulfate (SDS) sample loading buffer (100 mM Tris-HCl [pH 6.8], 4% SDS (NAKALAI TESQUE, Kyoto, Japan), 20% glycerol (FUJIFILM Wako Pure Chemical Corporation, Osaka, Japan), and 0.01% bromophenol blue (FUJIFILM Wako Pure Chemical Corporation, Osaka, Japan). Next, the beads were boiled for 5 min at 95 °C. For the Western blotting investigation, the cell lysate and precipitated proteins were separated using SDS-PAGE.

### 2.9. Western Blotting (WB)

All samples, including cell lysate from transfected HEK293 cells and precipitated proteins, were loaded into 14% SDS-PAGE gels. The proteins were then transferred to polyvinylidene difluoride membrane (Cytiva, Tokyo, Japan). The membranes were blocked with shaking for 1 h at room temperature using 0.5% [*w*/*v*] skim milk (NAKALAI TESQUE, Kyoto, Japan) in Tween-20 (+) (FUJIFILM Wako Pure Chemical Corporation, Osaka, Japan) Tris Buffered Saline (TBS-T; 100 mM Tris-HCl [pH 8.0], and 150 mM NaCl, 0.5% [*v*/*v*] Tween-20) and incubated with primary antibodies with shaking overnight at 4 °C. Subsequently, the membranes were incubated with HRP-conjugated secondary antibody with shaking at room temperature for 1 h after three washes in TBS-T. The immunoreactive proteins were developed using an enhanced chemiluminescence kit (Thermo Fisher Scientific, Waltham, MA, USA) and detected using the FUSION FX imaging system version 7 (Vilber Lourmat, Collégien, Seine-et-Marne, France).

### 2.10. HiBiT Lytic Detection System

Following transfection, the culture media (CM) were gathered and centrifuged for 5 min at 1200 rpm, and 100 µL of supernatant was transferred into black glass bottom 96-well cell culture plates (61-9713-47 EZVIEW). Each well was filled with 100 µL of DMEM (10% FBS) to prepare the cell lysates. The confluent cells were detached from the surface and lysed with NanoGlo HiBiT Lytic Reagent (Promega, Madison, WI, USA N3030, a buffer containing the recombinant N-terminus of Nano luciferase (LgBiT) and Nano luciferase substrate Furimazine). The luminescence intensity of untreated DMEM (10% FBS) was set as a background. The luminescence intensity of HTT-HiBiT [Htt-PolyQ72-Hibit-opt_pcDNA3.1(+))] (manufactured by GenScript, Piscataway, NJ, USA; Supplementary Figure S2) was measured in a plate by adding the same volume of NanoGlo HiBiT Lytic Reagent to each well. The reagent-treated cell lysates and culture media were incubated for a 10 min rotation. Then, the luminescence intensities of the cell lysate and culture medium were evaluated using a microplate reader (BioTek, Winooski, VT, USA) at a one-second integration time. All cell lysate and culture media’s luminescence intensities were adjusted by deducting the background’s luminescence intensity.

### 2.11. Immunocytochemistry (ICC) Staining

The culture medium was first removed, and the cells were washed with ice-cold PBS. Subsequently, the cells were fixed in ice-cold 100% methanol for 5 min and then washed three times with ice-cold PBS. Blocking was carried out using 1% bovine serum albumin (BSA) diluted in PBS for 1 h. The primary antibody was applied and incubated overnight at 4 °C. Following incubation, the primary antibody solution was decanted, and the cells were washed three times with PBS, with each wash lasting 5 min. The secondary antibody was then incubated for 1 h in the dark, diluted in 1% BSA in PBS at room temperature. Nuclear staining was performed using 4′,6-diamidino-2-phenylindole (DAPI, Dojindo, Kumamoto, Japan). After decanting the secondary antibody solution, the cells were washed three times with PBS, with each wash lasting 5 min and performed in the dark. The cells were subsequently mounted with VECTASHIELD mounting medium (Vector Laboratories). Confocal imaging was conducted using a 63× objective lens on a confocal laser-scanning microscope (Leica TCS SP8, Wetzlar, Germany).

### 2.12. Statistical Analysis

The measurement data were analyzed using GraphPad Prism 7.0 (GraphPad Software, LaJolla, CA, USA) statistical software and expressed as mean ± SD (standard deviation). The differences between two groups were calculated using two-sided Welch’s *t* test without paired data. A *p*-value of <0.05 was considered statistically significant.

## 3. Results

### 3.1. UBL3-Positive Inclusions Are Found in the Cytoplasm and Nuclei of Neurons Within the Striatum of HD Patients

To determine whether UBL3 is associated with the HD pathological process, we first conducted IHC staining using postmortem brain tissues from HD patients with the anti-polyglutamine antibody (1C2) and the anti-UBL3 antibody. Although HD is increasingly recognized as a neurodegenerative disorder affecting both the brain and the entire body, the striatum are the initial and most vulnerable areas affected by the mutant HTT gene in HD patients [20,21]. Therefore, we chose to perform staining in the striatum region to investigate any specific UBL3 distribution patterns.

**Figure 1:**
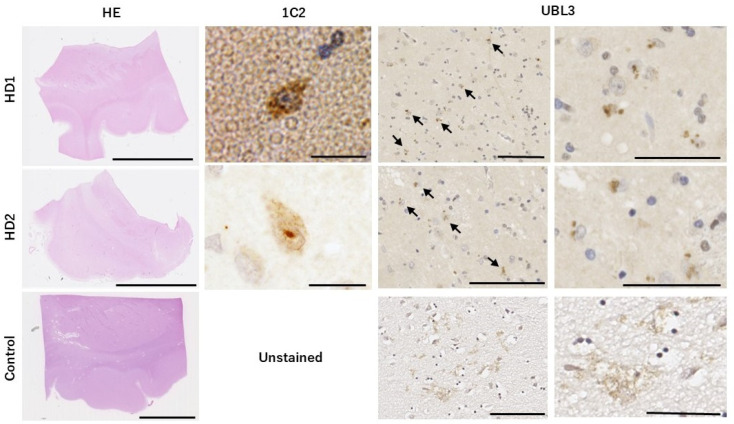
Distribution of UBL3 in the striatum site of postmortem brain tissue in Huntington patients and control (Scale bars: HE-staining 10 mm, 1C2-staining 10 μm, UBL3 left 100 μm and right 50 μm, Arrows: UBL3-Positive Inclusions) The first column provides an overview of hematoxylin- and eosin (HE)-stained striatum regions of the brain. The second column shows the presence of polyglutamine in the nuclei of neurons. Subsequent staining with the UBL3 antibody reveals that UBL3 is distributed as inclusions in the cytoplasm and nuclei of neurons within the striatum of HD patients. In contrast, UBL3 exhibits a more diffuse, dot-like distribution in the striatum of the control sample.

The results of the HE staining provided an overview of the brain regions (Figure 1). Examination with the anti-polyglutamine antibody (1C2) suggested the presence of polyglutamine accumulated within the nuclei of neurons. Anti-UBL3 antibody staining revealed that UBL3 was present as an inclusion body in the cytoplasm and nuclei of the neurons, which were sparsely distributed throughout the striatum of HD patients. In contrast, UBL3 exhibited a rather diffuse dot-like distribution in the striatum of the control samples. The abnormal presence of UBL3 in the neurons of HD patients suggests that UBL3 may indeed be involved in the pathophysiological processes associated with HD development.

### 3.2. UBL3 Interacts with Expanded polyQ-Containing N-Terminal HTT Fragments

It has been observed that the N-terminal fragment of mutant HTT, which includes exon1 with the expanded polyQ chain, represents the primary toxic and pathogenic species [22,23]. Those expanded polyQ-containing N-terminal HTT fragments have a strong tendency to aggregate and exhibit toxic properties, ultimately leading to neuronal dysfunction and death [24,25]. Moreover, recent research has indicated that these expanded polyQ-containing N-terminal HTT fragments are accessible to sEVs, contributing to their involvement in HD pathology [26,27]. Therefore, we formulated a hypothesis suggesting that UBL3 might interact with these expanded polyQ-containing N-terminal HTT fragments.

**Figure 2:**
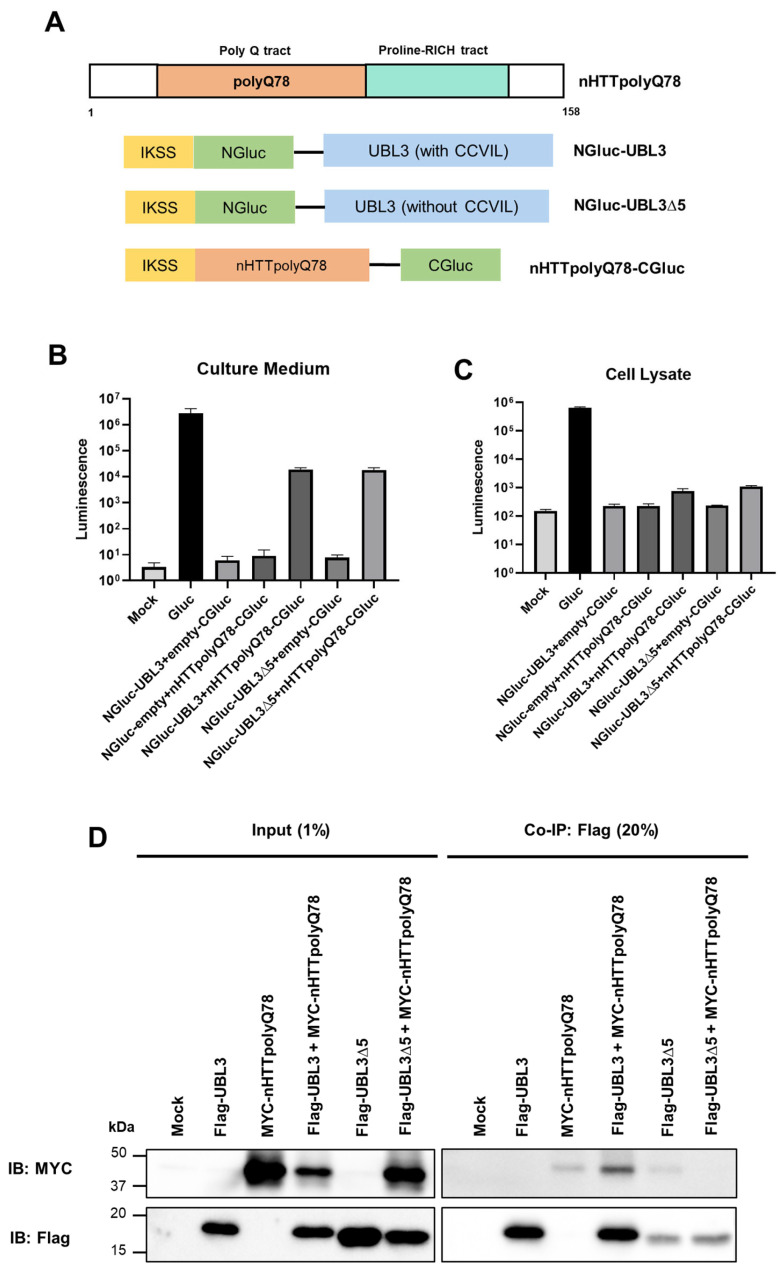
UBL3 interacts with expanded polyglutamine-containing N-terminal huntingtin fragments: (**A**) Schematic of expanded polyglutamine-containing N-terminal huntingtin fragments (nHTTpolyQ78) and split Gluc-tagged proteins, including NGluc-UBL3, NGluc-UBL3∆5, and nHTTpolyQ78-CGluc. (**B**,**C**) Luminescence of the culture medium and cell lysate from the transfected HEK293 cells with various combinations. The luminescence values are presented as mean ± S.D. from three independent experiments. (**D**) Co-immunoprecipitated Flag-UBL3 and Flag-UBL3∆5 interact with MYC-nHTTpolyQ78. The input lanes represent 1% of the sample before Co-IP, and the Co-IP lanes represent 20% of the Co-IP products.

To confirm the interaction between UBL3 and expanded polyQ-containing N-terminal HTT fragments, we initially utilized a Gaussia princeps-based split-luciferase complementation assay (Gluc SLCA). SLCA is a powerful technique that involves reassembling the N-terminal and C-terminal fragments of Gluc, enabling the detection of protein–protein interactions in vitro [28]. We constructed thenHTTpolyQ78-CGluc plasmid by fusing the expanded polyQ-containing N-terminal HTT fragments and C-terminal fragment of Gluc (Figure 2A). In our previous study, we reported that the C-terminal cysteine motif of UBL3, CCVIL, is a key site for post-translational modification and contains a membrane anchoring the CAAX motif [4]. So, we also introduced UBL3∆5, UBL3, with a C-terminal cysteine motif deletion mutation, to see if this motif would affect the interaction of UBL3 with nHTTpolyQ78. Subsequently, we co-expressed various combinations of SLCA constructs in HEK293 cells and measured the luciferase intensity in both the cell culture medium and cell lysate. Notably, strong luminescence intensities were observed in both fractions for two groups: the NGluc-UBL3 with the nHTTpolyQ78-CGluc group and the NGluc-UBL3∆5 with the nHTTpolyQ78-CGluc group (Figure 2B,C). In the group-overexpression Gluc, we observed very intense luminescence in both the cell culture medium and the cell lysates, while the results from the remaining control groups did not significantly differ from the background level.

To further validate the interaction of UBL3 and nHTTpolyQ78, we constructed an MYC-tagged nHTTpolyQ78 plasmid and transfected it into HEK293 cells in combination with Flag-UBL3 and Flag-UBL3∆5, respectively. The signal of MYC-nHTTpolyQ78 was detected from the co-IP of Flag-UBL3 but not in the co-IP of Flag-UBL3∆5 (Figure 2D).

### 3.3. UBL3 Alters the Sorting of Expanded polyQ-Containing N-Terminal HTT Fragments Within Cells

**Figure 3:**
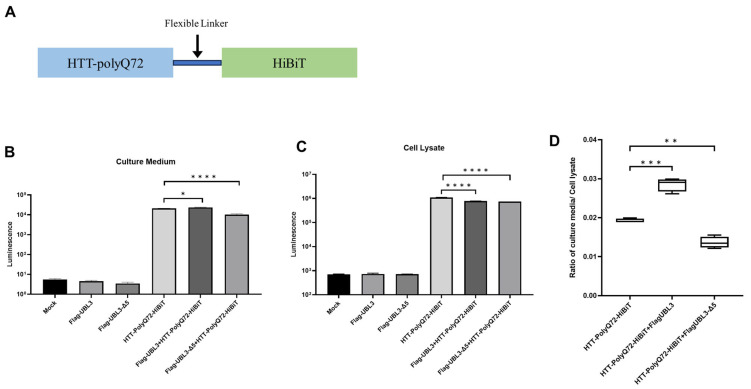
UBL3 alters the intracellular sorting of expanded polyglutamine-containing N-terminal huntingtin fragments (* *p* < 0.05, ** *p* < 0.01, *** *p* < 0.001, *** *p* < 0.0001): (**A**) Schematic of mHTT-HiBiT. (**B**,**C**) Luminescence of the culture medium and cell lysate from the transfected HEK293 cells with various combinations. The luminescence ± SD in quadruplicate experiments is shown. (**D**) Relative ratio of luminescence of culture media divided by cell lysate.

To further validate our findings from the Gluc SLCA, we generated a HiBiT-tagged HTT (HTT-HiBiT) construct to investigate the intracellular behavior of mHTT using the HiBiT lytic detection system. This is a highly sensitive bioluminescent-protein quantification tool used for a range of applications, including protein–protein interaction studies [29] and high-throughput screening [30]. We constructed the nHTT^PolyQ72-HiBiT plasmid by fusing the N-terminal region of huntingtin (HTT) with an expanded polyQ^72 tract to the C-terminal of HiBiT, connected via a flexible linker (Figure 3A). In the UBL3-HTT^PolyQ72 co-transfected group, we observed a significant increase in luminescence in the culture medium, accompanied by a decrease in the cell lysate (Figure 3B,C). These results suggest that UBL3 promotes the secretion of mHTT into the extracellular space. In contrast, in the UBL3∆5-HTT^PolyQ72 co-transfected group, both the culture medium and cell lysate showed a significant decrease in luminescence, which likely indicates a modification in the sorting of HTT^PolyQ72, potentially pointing to a degradation pathway. The relative ratios further confirmed an increase in UBL3-HTT^PolyQ72 levels in the culture medium as compared to cell lysate, supporting the idea that UBL3 promotes the secretion of mHTT from the cell (Figure 3D).

### 3.4. UBL3 Colocalizes with Expanded polyQ-Containing N-Terminal HTT Fragments and Affects Their Distribution Within Cells

**Figure 4:**
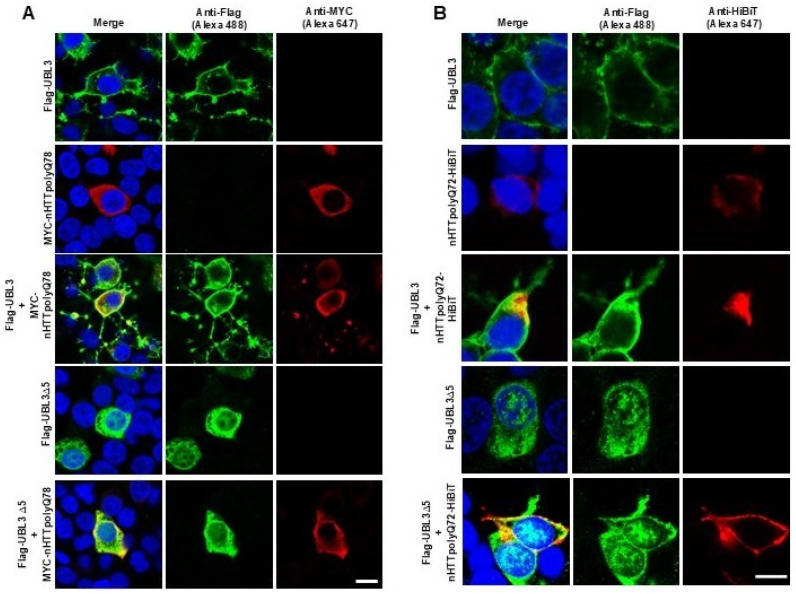
UBL3 colocalizes with expanded polyglutamine-containing N-terminal huntingtin fragments (Scale bars: 10 μm): (**A**) Immunocytochemistry (ICC)-staining representative images of HEK293 cells transfected or co-transfected with Flag-UBL3, Flag-UBL3∆5, and MYC-HTTpolyQ78. (**B**) Immunocytochemistry (ICC)-staining representative images of HEK293 cells transfected or co-transfected with Flag-UBL3, Flag-UBL3∆5, and nHTTpolyQ72-HiBiT.

We performed immunocytochemical (ICC) staining to observe the colocalization of UBL3 and nHTTpolyQ, aiming to investigate whether co-transfection affects their distribution patterns. An anti-Flag antibody was used to detect UBL3, an anti-MYC antibody for nHTTpolyQ78, and an anti-HiBiT antibody for nHTTpolyQ72. The results revealed that Flag-UBL3 was predominantly localized at the cell periphery, while Flag-UBL3∆5 displayed a diffuse distribution throughout the cytoplasm and nuclei (Figure 4A,B). When MYC-nHTTpolyQ78 or nHTTpolyQ72-HiBiT was co-transfected with Flag-UBL3, both exhibited a colocalization pattern at the cell periphery. Notably, similar colocalization patterns were also observed in cells co-transfected with Flag-UBL3∆5 and either MYC-nHTTpolyQ78 or nHTTpolyQ72-HiBiT.

## 4. Discussion

We visualized the distribution of UBL3 in postmortem brain tissue, specifically within the neurons of the striatum in Huntington’s disease (HD) patients, and identified its interaction with polyQ-expanded huntingtin fragments. These findings provide valuable insights into the pathophysiological processes underlying HD and suggest new strategies for its diagnosis and treatment.

In HD pathology, the most vulnerable and earliest-affected cells are the predominant GABAergic spiny projection neurons of the striatum, known as medium spiny neurons (MSNs) [31,32]. The degeneration and loss of MSNs are hallmark features of HD [33], leading to a hyperkinetic state and the onset of involuntary movements and chorea [34]. The presence of UBL3-positive inclusions in the cytoplasm and nuclei of striatal neurons in HD patients raises the possibility that UBL3 may play a role in the pathological process of MSN degeneration in HD. Certainly, this still needs to be further explored.

The 17 amino acids at the N-terminus of mutant HTT are subject to various post-translational modifications, including phosphorylation and acetylation, which influence the aggregation and subcellular localization of the protein [35,36]. These N-terminal amino acids of nHTTpolyQ78 may serve as the site for UBL3’s interaction with nHTTpolyQ78. In our previous study, we reported that the functionality of UBL3 in post-translational modification relies on its C-terminal cysteine motif [2]. However, in the current study, we observed that a deletion mutation of this cysteine motif at the C-terminus of UBL3 did not affect its interaction with nHTTpolyQ78, as shown in the Gluc SLCA. We speculate that the interactions between UBL3 and the expanded polyQ-containing N-terminal HTT fragments may be mediated by both specific motifs and mechanisms that do not involve motifs. Due to the high sensitivity and wide dynamic range of the split luciferase assay, luciferase intensity can be detected even when UBL3, carrying a deletion mutation in its C-terminal cysteine motif, indirectly interacts with nHTTpolyQ78 within the cell.

Our cell lysate results from the Gluc SLCA did not show a strong fusion of UBL3 and nHTTpolyQ78, although Co-IP indicated an interaction between them. This discrepancy can be explained by the presence of NaCl in the detergent, which can promote the extraction of proteins, leading to a positive interaction in the Co-IP assay. The weak signal of Flag-UBL3∆5 in the Co-IP is likely due to the efficiency of the transfection.

When constructing the plasmid for the HTT-HiBiT detection assay, we intentionally excluded the IKSS region from the sequence to allow for a more accurate assessment of UBL3’s secretion capability upon mHTT. Consequently, the luminescence measured in the culture medium during this assay was lower than that observed in the Gluc SLCA, though it was still sufficient to demonstrate secretion promotion by UBL3. For UBL3∆5, the luminescence decreased in both the culture medium and the cell lysate, suggesting alterations in the sorting of HTT-PolyQ72, possibly indicating a decomposition pathway.

Our ICC-staining results further demonstrate that deletion mutations in the C-terminal cysteine residue of UBL3 alter its intracellular distribution, resulting in a diffuse localization throughout the cytoplasm and nuclei. Notably, colocalization was also observed at the cell periphery, where UBL3 may play a role in actively sorting mHTT into the extracellular space. These findings were consistent in both the MYC-nHTTpolyQ78 and nHTTpolyQ72-HiBiT assays. For UBL3∆5, there was no significant difference in colocalization between the two proteins as compared to UBL3, which may suggest the intracellular degradation pathway discussed in the previous paragraph. However, this hypothesis warrants further investigation in future research.

The primary strategy for treating Huntington’s disease focuses on reducing mHTT levels, which has been reported to improve motor performance, cognitive function, and overall survival [37,38,39]. Several potential therapeutic approaches that align with this strategy include DNA-targeting techniques, small-molecule splicing modulators, clearance of mutant HTT, and other strategies addressing inflammation and cell replacement [40,41,42]. However, it is important to note that there are currently no approved treatments capable of altering the course of HD. Much research has been conducted on the post-translational modifications (PTMs) of mutant huntingtin (mHTT), including SUMOylation [43] and ubiquitination [44], and their potentials as targets for novel therapies. Our discovery of the interaction between UBL3 and mHTT offers new insights into the PTM landscape of mHTT. Additionally, our previous studies demonstrated that the EGFR pathway inhibitor osimertinib reduces the interaction between UBL3 and α-synuclein [4], while MGST3 enhances this interaction [6], leading to the increased extracellular transport of α-synuclein under oxidative stress conditions. Future experiments, particularly under drug-treated or oxidative stress conditions, are needed to explore new therapeutic approaches targeting the UBL3-mHTT interaction, with the aim of reducing the levels of the toxic protein.

## 5. Conclusions

Our results demonstrate that UBL3 interacts with polyQ-expanded mutant huntingtin (mHTT) fragments, which colocalize within the cell and influence their intracellular sorting. Overall, our findings provide new insights into the pathology of Huntington’s disease and suggest that modulation of the UBL3 pathway may serve as a potential therapeutic target in the near future.

## 6. Limitation

Our current experimental methods employ a newly developed tagging system. However, incorporating a direct interaction assay, such as the Proximity Ligation Assay, would enhance our findings. While we used HEK293 cells throughout our experiments, results from primary neurons would provide a more relevant representation of Huntington’s disease. Additionally, using full-length mHTT as a positive control would further strengthen our research.

## Figures and Tables

**Table 1:** Characteristics of subjects from whom postmortem brain samples were obtained.

Clinical Diagnosis	Age	Sex	PMI ^1^	Cause of Death	PolyQ Repeat No. (Pathogenic, Normal)
Hungtinton’s Disease (HD 1)	57	M	2 h 37 m	Respiratory Failure	(50, 22)
Hungtinton’s Disease (HD 2)	53	F	12 h 17 m	Respiratory Failure	(53, 20)
Non-Hodgkin’s Lymphoma (Control)	68	M	4 h 18 m	Gastrointestinal Hemorrhage	Untested

^1^ PMI: postmortem interval, the time that has elapsed since the person has died.

## Data Availability

All relevant data were reported within the article. Further supporting data will be provided upon a written request addressed to the corresponding author.

## Supplementary Materials

The following supporting information can be downloaded at https://www.mdpi.com/article/10.3390/neurolint16060089/s1: Figure S1: Immunoblotting and immunohistochemical staining results of the polyclonal anti-UBL3 antibody; Figure S2: Schematic and sequence of nHTTpolyQ72-HiBiT.

## References

[1] Downes B.P., Saracco S.A., Lee S.S., Crowell D.N., Vierstra R.D. (2006). MUBs, a Family of Ubiquitin-fold Proteins That Are Plasma Membrane-anchored by Prenylation. J. Biol. Chem..

[2] Ageta H., Ageta-Ishihara N., Hitachi K., Karayel O., Onouchi T., Yamaguchi H., Kahyo T., Hatanaka K., Ikegami K., Yoshioka Y. (2018). UBL3 modification influences protein sorting to small extracellular vesicles. Nat. Commun..

[3] Takanashi Y., Kahyo T., Kamamoto S., Zhang H., Chen B., Ping Y., Mizuno K., Kawase A., Koizumi K., Satou M. (2022). Ubiquitin-like 3 as a new protein-sorting factor for small extracellular vesicles. Cell Struct. Funct..

[4] Chen B., Hasan M., Zhang H., Zhai Q., Waliullah A.S.M., Ping Y., Zhang C., Oyama S., Mimi M.A., Tomochika Y. (2023). UBL3 Interacts with Alpha-Synuclein in Cells and the Interaction Is Downregulated by the EGFR Pathway Inhibitor Osimertinib. Biomedicines.

[5] Yan J., Zhang H., Tomochika Y., Chen B., Ping Y., Islam S., Aramaki S., Sato T., Nagashima Y., Nakamura T. (2023). UBL3 Interaction with α-Synuclein Is Downregulated by Silencing MGST3. Biomedicines.

[6] Yan J., Kahyo T., Zhang H., Ping Y., Zhang C., Jiang S., Ji Q., Ferdous R., Islam S., Oyama S. (2024). Alpha-Synuclein Interaction with UBL3 Is Upregulated by Microsomal Glutathione S-Transferase 3, Leading to Increased Extracellular Transport of the Alpha-Synuclein under Oxidative Stress. Int. J. Mol. Sci..

[7] McColgan P., Tabrizi S.J. (2018). Huntington’s disease: A clinical review. Eur. J. Neurol..

[8] Nopoulos P.C. (2016). Huntington disease: A single-gene degenerative disorder of the striatum. Dialogues Clin. Neurosci..

[9] Macdonald M. (1993). A novel gene containing a trinucleotide repeat that is expanded and unstable on Huntington’s disease chromosomes. Cell.

[10] Palaiogeorgou A.M., Papakonstantinou E., Golfinopoulou R., Sigala M., Mitsis T., Papageorgiou L., Diakou I., Pierouli K., Dragoumani K., Spandidos D.A. (2023). Recent approaches on Huntington’s disease (Review). Biomed. Rep..

[11] Bauer P.O., Nukina N. (2009). The pathogenic mechanisms of polyglutamine diseases and current therapeutic strategies. J. Neurochem..

[12] Bañez-Coronel M., Porta S., Kagerbauer B., Mateu-Huertas E., Pantano L., Ferrer I., Guzmán M., Estivill X., Martí E. (2012). A Pathogenic Mechanism in Huntington’s Disease Involves Small CAG-Repeated RNAs with Neurotoxic Activity. PLoS Genet..

[13] Liu L., Tong H., Sun Y., Chen X., Yang T., Zhou G., Li X.J., Li S. (2023). Huntingtin Interacting Proteins and Pathological Implications. Int. J. Mol. Sci..

[14] Zhao T., Hong Y., Li S., Li X.-J. (2016). Compartment-Dependent Degradation of Mutant Huntingtin Accounts for Its Preferential Accumulation in Neuronal Processes. J. Neurosci..

[15] Steffan J.S., Agrawal N., Pallos J., Rockabrand E., Trotman L.C., Slepko N., Illes K., Lukacsovich T., Zhu Y.-Z., Cattaneo E. (2004). SUMO Modification of Huntingtin and Huntington’s Disease Pathology. Science.

[16] O’rourke J.G., Gareau J.R., Ochaba J., Song W., Raskó T., Reverter D., Lee J., Monteys A.M., Pallos J., Mee L. (2013). SUMO-2 and PIAS1 modulate insoluble mutant huntingtin protein accumulation. Cell Rep..

[17] Mishra A., Dikshit P., Purkayastha S., Sharma J., Nukina N., Jana N.R. (2008). E6-AP Promotes Misfolded Polyglutamine Proteins for Proteasomal Degradation and Suppresses Polyglutamine Protein Aggregation and Toxicity. J. Biol. Chem..

[18] Mutant Huntingtin Stalls Ribosomes and Represses Protein Synthesis in a Cellular Model of Huntington Disease|Nature Communications. https://www.nature.com/articles/s41467-021-21637-y.

[19] Reilmann R., Leavitt B.R., Ross C.A. (2014). Diagnostic criteria for Huntington’s disease based on natural history. Mov. Disord..

[20] Vonsattel J.P.G., DiFiglia M. (1998). Huntington Disease. J. Neuropathol. Exp. Neurol..

[21] Mätlik K., Baffuto M., Kus L., Deshmukh A.L., Davis D.A., Paul M.R., Carroll T.S., Caron M.-C., Masson J.-Y., Pearson C.E. (2024). Cell-type-specific CAG repeat expansions and toxicity of mutant Huntingtin in human striatum and cerebellum. Nat. Genet..

[22] Bates G.P., Dorsey R., Gusella J.F., Hayden M.R., Kay C., Leavitt B.R., Nance M., Ross C.A., Scahill R.I., Wetzel R. (2015). Huntington disease. Nat. Rev. Dis. Primer.

[23] Yang H., Yang S., Jing L., Huang L., Chen L., Zhao X., Yang W., Pan Y., Yin P., Qin Z.S. (2020). Truncation of mutant huntingtin in knock-in mice demonstrates exon1 huntingtin is a key pathogenic form. Nat. Commun..

[24] DiFiglia M., Sapp E., Chase K.O., Davies S.W., Bates G.P., Vonsattel J.P., Aronin N. (1997). Aggregation of Huntingtin in Neuronal Intranuclear Inclusions and Dystrophic Neurites in Brain. Science.

[25] Schilling G., Klevytska A., Tebbenkamp A.T.N., Juenemann K., Cooper J., Gonzales V., Slunt H., Poirer M., Ross C.A., Borchelt D.R. (2007). Characterization of Huntingtin Pathologic Fragments in Human Huntington Disease, Transgenic Mice, and Cell Models. J. Neuropathol. Exp. Neurol..

[26] Ananbeh H., Vodicka P., Skalnikova H.K. (2021). Emerging Roles of Exosomes in Huntington’s Disease. Int. J. Mol. Sci..

[27] Deng J., Koutras C., Donnelier J., Alshehri M., Fotouhi M., Girard M., Casha S., McPherson P.S., Robbins S.M., Braun J.E.A. (2017). Neurons Export Extracellular Vesicles Enriched in Cysteine String Protein and Misfolded Protein Cargo. Sci. Rep..

[28] Wille T., Blank K., Schmidt C., Vogt V., Gerlach R.G. (2012). Gaussia princeps Luciferase as a Reporter for Transcriptional Activity, Protein Secretion, and Protein-Protein Interactions in Salmonella enterica Serovar Typhimurium. Appl. Environ. Microbiol..

[29] Arakawa M., Morita E. (2023). Protein Pull-down Assay Using HiBiT-tag-dependent Luciferase Activity Measurement. Bio-Protocol.

[30] Chen Y., Lear T.B., Evankovich J.W., Larsen M.B., Lin B., Alfaras I., Kennerdell J.R., Salminen L., Camarco D.P., Lockwood K.C. (2021). A high-throughput screen for TMPRSS2 expression identifies FDA-approved compounds that can limit SARS-CoV-2 entry. Nat. Commun..

[31] Lanciego J.L., Luquin N., Obeso J.A. (2012). Functional Neuroanatomy of the Basal Ganglia. Cold Spring Harb. Perspect. Med..

[32] Conforti P., Bocchi V.D., Campus I., Scaramuzza L., Galimberti M., Lischetti T., Talpo F., Pedrazzoli M., Murgia A., Ferrari I. (2022). In vitro-derived medium spiny neurons recapitulate human striatal development and complexity at single-cell resolution. Cell Rep. Methods.

[33] Veldman M.B., Yang X.W. (2018). Molecular insights into cortico-striatal miscommunications in Huntington’s disease. Curr. Opin. Neurobiol..

[34] Plotkin J.L., Surmeier D.J. (2015). Corticostriatal synaptic adaptations in Huntington’s disease. Curr. Opin. Neurobiol..

[35] Cariulo C., Azzollini L., Verani M., Martufi P., Boggio R., Chiki A., Deguire S.M., Cherubini M., Gines S., Marsh J.L. (2017). Phosphorylation of huntingtin at residue T3 is decreased in Huntington’s disease and modulates mutant huntingtin protein conformation. Proc. Natl. Acad. Sci. USA.

[36] Bassi S., Tripathi T., Monziani A., Di Leva F., Biagioli M., Delgado-Morales R. (2017). Epigenetics of Huntington’s Disease. Neuroepigenomics in Aging and Disease.

[37] Zeitler B., Froelich S., Marlen K., Shivak D.A., Yu Q., Li D., Pearl J.R., Miller J.C., Zhang L., Paschon D.E. (2019). Allele-selective transcriptional repression of mutant HTT for the treatment of Huntington’s disease. Nat. Med..

[38] Kordasiewicz H.B., Stanek L.M., Wancewicz E.V., Mazur C., McAlonis M.M., Pytel K.A., Artates J.W., Weiss A., Cheng S.H., Shihabuddin L.S. (2012). Sustained Therapeutic Reversal of Huntington’s Disease by Transient Repression of Huntingtin Synthesis. Neuron.

[39] Southwell A.L., Kordasiewicz H.B., Langbehn D., Skotte N.H., Parsons M.P., Villanueva E.B., Caron N.S., Østergaard M.E., Anderson L.M., Xie Y. (2018). Huntingtin suppression restores cognitive function in a mouse model of Huntington’s disease. Sci. Transl. Med..

[40] Tabrizi S.J., Ghosh R., Leavitt B.R. (2019). Huntingtin Lowering Strategies for Disease Modification in Huntington’s Disease. Neuron.

[41] Tabrizi S.J., Estevez-Fraga C., van Roon-Mom W.M.C., Flower M.D., I Scahill R., Wild E.J., Muñoz-Sanjuan I., Sampaio C., E Rosser A., Leavitt B.R. (2022). Potential disease-modifying therapies for Huntington’s disease: Lessons learned and future opportunities. Lancet Neurol..

[42] Pan L., Feigin A. (2021). Huntington’s Disease: New Frontiers in Therapeutics. Curr. Neurol. Neurosci. Rep..

[43] Soares E.S., Prediger R.D., Brocardo P.S., Cimarosti H.I. (2022). SUMO-modifying Huntington’s disease. IBRO Neurosci. Rep..

[44] Fiorillo A., Morea V., Colotti G., Ilari A. (2021). Huntingtin Ubiquitination Mechanisms and Novel Possible Therapies to Decrease the Toxic Effects of Mutated Huntingtin. J. Pers. Med..

