# Supplementary material for: UBL3 Interacts with PolyQ-Expanded Huntingtin Fragments and Modifies Their Intracellular Sorting
Source: Neurol Int. 2024 Oct 22;16(6):1175–88. doi: 10.3390/neurolint16060089 (PMC11503352; doi:10.3390/neurolint16060089)
Supplement: Supplementary file 1 [file neurolint-3157650-supplementary.pdf › neurolint-3157650-supplementary.pdf]

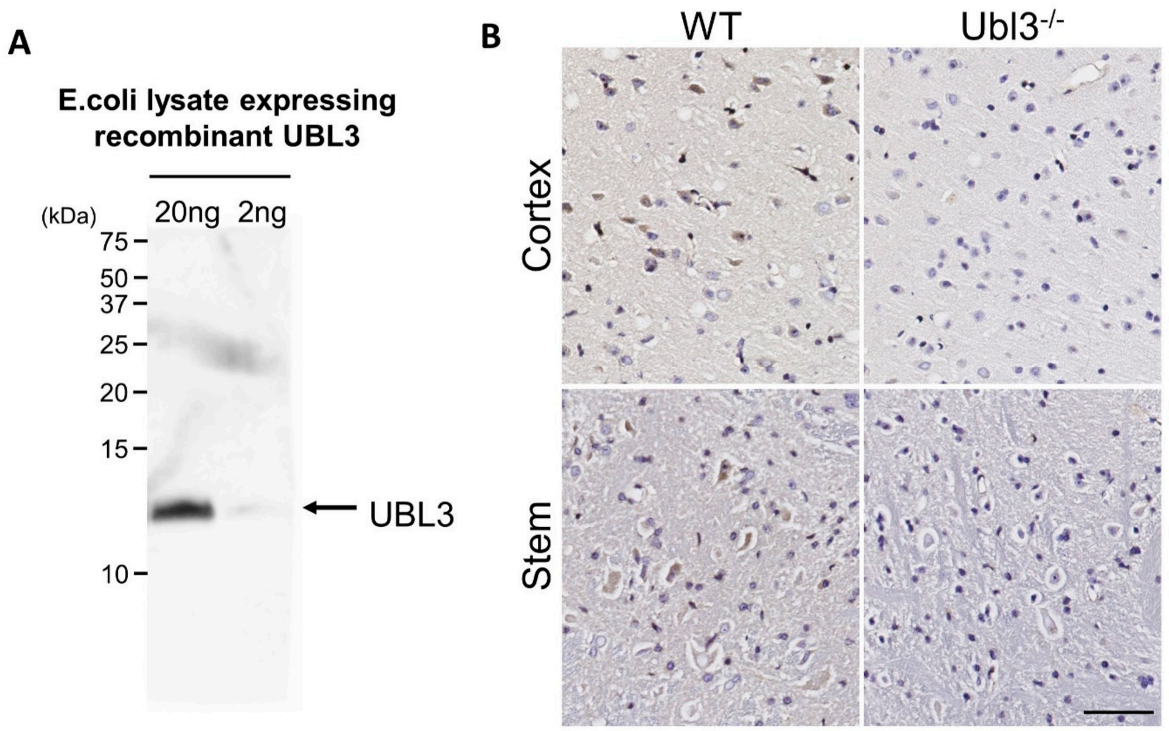

Figure S1. Immunoblotting and immunohistochemical staining results obtained by using the polyclonal anti-UBL3 antibody (Scale bars, 50 μm WT: wild type; Ubl3-/-: Ubl3 knockout):
(A) immunoblotting results using E. coli lysates expressing recombinant UBL3, including 20 ng/lane and 2 ng/lane, and (B) immunohistochemical staining results in WT and Ubl3-/- mice brains.

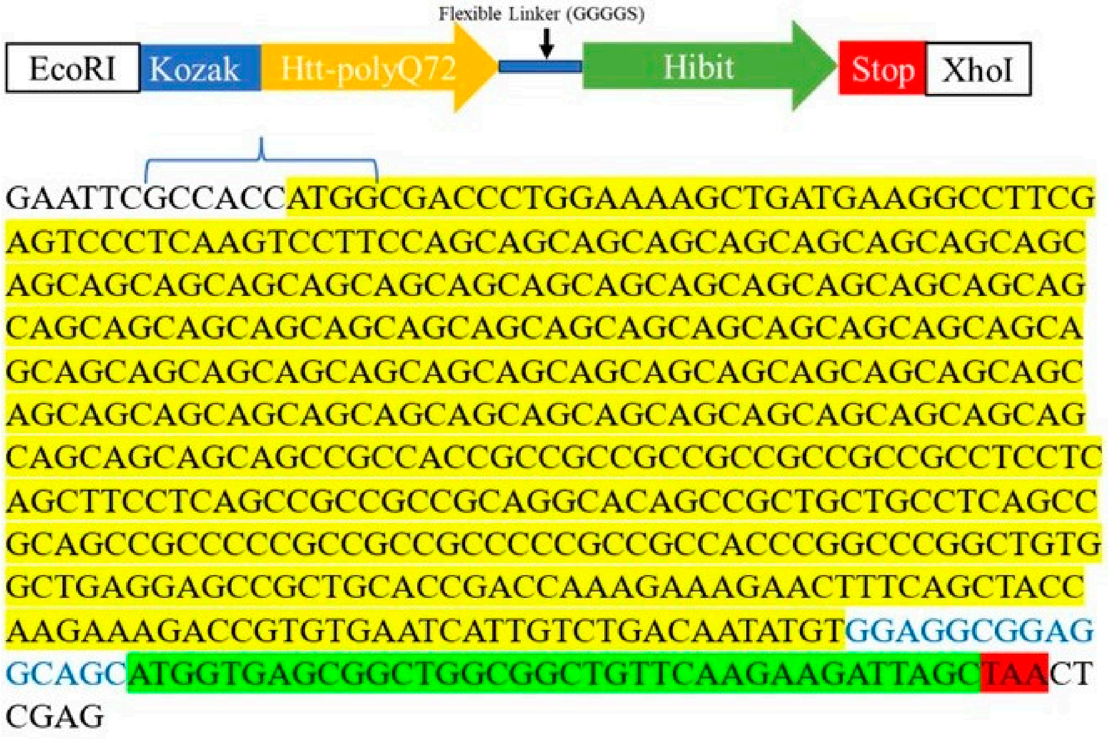

Figure S2. Schematic and sequence of nHTTpolyQ72-HiBiT.
